# Protein abundance inference via expectation-maximization in fluorosequencing

**DOI:** 10.1093/bioadv/vbag053

**Published:** 2026-02-15

**Authors:** Javier Kipen, Matthew Beauregard Smith, Thomas Blom, Sophia Bailing Zhou, Edward M Marcotte, Joakim Jaldén

**Affiliations:** Department of Intelligent Systems, Division of Information Science and Engineering, KTH Royal Institute of Technology, 114 28 Stockholm, Sweden; Erisyon, Inc., Austin, TX 78752, United States; Erisyon, Inc., Austin, TX 78752, United States; Technical University of Munich, Munich 80333, Germany; Department of Molecular Biosciences, The University of Texas at Austin, Austin, TX 78712, United States; Department of Intelligent Systems, Division of Information Science and Engineering, KTH Royal Institute of Technology, 114 28 Stockholm, Sweden

## Abstract

**Summary:**

Fluorosequencing generates millions of single peptide reads, yet a principled route to quantitative protein abundances has been lacking. We present a probabilistic framework that adapts expectation–maximization (EM) to the fluorosequencing measurement process, using posterior peptide probabilities from existing classifiers to estimate relative protein abundances. The algorithm iteratively updates abundances to maximize the likelihood of observed reads. We first evaluate five-protein simulations with realistic labeling and system errors. A simple Python implementation processes one million reads in under ten seconds on a standard workstation and reduces the mean absolute error by over an order of magnitude relative to a uniform-abundance guess, indicating robust performance in small-scale settings. We also assess scalability with full human-proteome simulations (20 642 proteins). Ten million reads are processed in under four hours on an NVIDIA DGX with a single Tesla V100 GPU, confirming tractability at proteome scale. Under current fluorosequencing error rates, the method yields modest accuracy gains, but when error rates are reduced, estimation error drops markedly, indicating that chemistry improvements would translate directly into more accurate quantitative proteomics. Overall, EM-based inference provides a scalable, model-driven bridge from peptide-level classification to protein-level quantification in fluorosequencing. Furthermore, the framework can also serve as a refinement step within other inference methods.

**Availability and implementation:**

The code and data utilized to produce all the results of this paper is at https://github.com/JavierKipen/ProtInfGPU.

## 1 Introduction

Next-generation sequencing has transformed genetic analysis, enabling rapid, low-cost whole-genome sequencing (Eren *et al*. 2022). In contrast, protein sequencing technologies have lagged behind, despite proteins being the primary functional molecules in cells and often more directly indicative of biological state. Many biological and medical applications, from cancer biomarker discovery to the analysis of complex signaling pathways, rely not only on identifying proteins but also on quantifying their abundances with high precision. Existing approaches, such as mass spectrometry and affinity-based assays, have made considerable progress, yet they often struggle to simultaneously achieve high sensitivity, digital quantification, and multiplexed measurement within a single platform (Timp and Timp 2020).

Single-molecule protein sequencing (SMPS) seeks to address this gap (Restrepo-Pérez *et al*. 2018, Alfaro *et al*. 2021). Inspired by next-generation DNA sequencing, these techniques aim to identify and quantify individual protein molecules directly, without amplification, and at scale. While recent advances in nanopore-based methods (Brinkerhoff *et al*. 2021, Zhang *et al*. 2021, Yu *et al*. 2023) and emerging commercial platforms (Reed *et al*. 2022) hold significant promise, fluorosequencing (Swaminathan *et al*. 2015, 2018, Mapes *et al*. 2023) offers a compelling SMPS method that combines amino-acid labeling, Edman degradation, and imaging to extract partial peptide sequence information.

Some of the recent progress in fluorosequencing includes estimations of experimental error rates (Smith *et al*. 2024), analysis and reduction of dye-dye interactions (Bachman *et al*. 2022) and algorithms for classifying peptides from fluorescence readouts (Kipen and Jaldén 2023, Smith *et al*. 2023). These latter methods are used as algorithmic components in this paper.

Although protein inference is well studied for mass spectrometry (Nesvizhskii *et al*. 2003, Huang *et al*. 2012, The *et al*. 2016, Audain *et al*. 2017), analogous methods for fluorosequencing remain underexplored. In the case of mass spectrometry, protein inference is often considered independently of protein quantification, although some work has considered both at once, e.g. Spivak *et al*. (2012); Winkler (2021); He *et al*. (2016). An important distinction for fluorosequencing is that, as a single-molecule sequencing technique, identification and quantification are directly coupled, with each identification corresponding to one discrete peptide molecule in the sample, and hence an individual molecule of the source protein. We thus sought an integrated approach in which protein identifications, based on (single molecule) peptide sequences, were considered in the same statistical framework as their abundances.

In this work, we adapt Expectation-Maximization (EM), a well-established probabilistic inference technique, to fluorosequencing. We present a framework that estimates protein abundances from peptide-level posteriors, produced by peptide-level classifiers from Whatprot (Smith *et al*. 2023), Probeam (Kipen and Jaldén 2023) and an artificial oracle which has access to the true peptide. Here, “peptide posterior probability” denotes the model’s estimated likelihood that a read originates from a given peptide after observing the fluorescence data. This algorithm iteratively refines the estimates of protein abundances to maximize the likelihood of the observed fluorescence data. The approach is principled, computationally efficient, and scalable, aligning with the throughput potential of SMPS.

We validate the framework on simulated datasets, showing robust abundance recovery under realistic errors for small protein mixtures and tractable runtimes at proteome scale. Although improvements in proteome-wide abundance estimates are modest under current fluorosequencing error rates, our simulations demonstrate that reductions in these errors lead to markedly more accurate protein-level inference. While these results are encouraging, further methodological advances will be necessary to fully meet the demands of large-scale proteomic analysis in practical applications.

## 2 Method

### 2.1 Fluorosequencing

Fluorosequencing is a single-molecule protein sequencing (SMPS) technique inspired by methods used in DNA and RNA sequencing (Swaminathan *et al*. 2015, 2018). The process begins by denaturing proteins and proteolytically cleaving them into peptides, which are then chemically labeled with fluorescent dyes. Millions of these labeled peptides are immobilized in a flow cell and imaged using total internal reflection fluorescence (TIRF) microscopy.

Edman degradation is then performed in cycles, sequentially removing one N-terminal amino acid from each peptide. After each cycle, the fluorescence intensities of the peptides are measured. The pattern of fluorescence intensity drops across cycles is then analyzed by a peptide inference algorithm, which estimates the likelihoods of possible peptides generating that pattern. The possible peptides are known because the proteins in the sample, the labeling chemistry, and the proteolysis rules governing peptide cleavage are all defined.

Several systematic error sources affect inference, including incomplete Edman degradation, dye loss between cycles, and the non-observability of some peptides. These factors have been characterized previously (Smith *et al*. 2023, 2024).

Once peptide sequences and their probabilities are inferred from the fluorescence reads, they can be mapped back to proteins, enabling protein abundance estimation. This protein-level inference has not previously been described for fluorosequencing and is the focus of the present work.

### 2.2 Protein abundance inference in fluorosequencing

Let X denote the fluorosequencing dataset comprising $N_{r}$ reads. Each read $X_{k}$ is a matrix of fluorescence intensities with rows for dye channels and columns for Edman degradation cycles (observations x and $x_{k}$ denote realizations).

The reads $X_{k}$ are generated by different peptides attached to the wall. Certain peptides are mutually indistinguishable by fluorosequencing, and the indistinguishable groups are referred to as fluorescence strings. The number of possible fluorescence strings is finite and is determined by several factors: the proteins utilized, the enzymes used to digest the proteins (e.g. trypsin), and the specific amino acids labeled with fluorophores. We introduce the random variable *F*, representing a uniformly drawn fluorescence string in the experiment, with the domain of all possible fluorescence strings denoted as $D_{F}$. The distribution of *F* is given by $P_{F}(f)$ and peptides lacking labeled residues map to the null fluorescence string $f_{\text{null}}$.

The purpose of protein abundance inference is to be able to estimate the protein distribution present in a solution. Let $Y\in D_{Y}$ be a random variable representing a uniformly drawn protein, and $P_{Y}(y)$ the distribution of proteins, which is the target for estimation. Given that the number of possible proteins $N_{P}$ is known for our experiments, we can represent $P_{Y}(y)$ as $P_{Y}(y)=p_{y}$, where $p_{y}$ represents the relative abundance of the *y*th protein, and $\sum_{y}p_{y}=1$.

Given a known protein distribution $P_{Y}(y)$, the fluorescence string distribution $P_{F}(f)$ can be inferred a priori from known cleavage and labeling scheme.

The protein inference problem can be formulated as estimating the protein distribution that maximizes the likelihood of the observed dataset:


(1) $${\hat{P}}_{Y}=\underset{P_{Y}}{\text{argmax}}P_{X}(x)$$

Solving (1) is a highly complex problem, and in this paper, we present a method to obtain practical estimates ${\hat{P}}_{Y}$. While not necessarily optimal, the proposed method provides meaningful estimations and can be also used to refine other inference techniques.

### 2.3 Notation

To apply EM to the protein inference, we introduce auxiliary variables. A key challenge is that proteins and reads are not in one-to-one correspondence: each protein may generate multiple fluorescence strings, some unobservable. This mismatch violates standard EM assumptions on having the same number of hidden and observed variables, a problem that will be solved with our framework.

Let $F^{o}$ denote an *observable* fluorescence string; it shares the domain of *F* but satisfies $P_{F^{o}}(f_{\text{null}})=0$ where $f_{\text{null}}$ is the fluorescence string that represents no dyes on the string. The probability of dyes not attaching and the difference with $f_{\text{null}}$ results in $P_{F^{o}}(f)\neq P_{F}(f)$, and an example is provided in the Appendix, available as supplementary data at *Bioinformatics Advances* online to further clarify the variable $F^{o}$. However, we can compute $P_{F^{o}}(f)$ from a transformation of $P_{F}(f)$:


(2) $$P_{F^{o}}(f)=K(1-m^{N_{d}(f)})P_{F}(f)$$

where *m* is the dye miss rate, $N_{d}$ is a function that returns the number of amino acids that are ideally labeled and *K* is a normalization constant ($K^{-1}=\sum_{f\in D_{F}}\left[\left(1-m^{N_{d}(f)})P_{F}(f\right)\right]$). Notice that $N_{d}(f_{\text{null}})=0$, and then the probability of observing the null fluorescence string is zero too. The advantage of $F^{o}$ is that there is one realization per read in the dataset, and that $P_{X_{k}|F_{k}}(x_{k}|f)=P_{X_{k}|F_{k}^{o}}(x_{k}|f)$ as the fluorescence string itself is unchanged. Finally, we define $F_{k}^{o}$ as the experimentally observed fluorescence string that generated the *k*th read.

**Figure 1:**
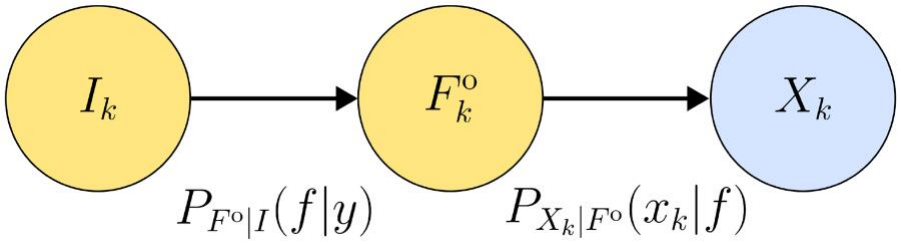
Relationship between the hidden protein indicator $I_{k}$, the hidden experimentally observable fluorescence string $F_{k}^{o}$ and the observable read $X_{k}$ for the *k*th read.

Let *I* be the *protein indicator*, i.e. the protein of origin for a read, with mass function $P_{I}(y)=q_{y}$. For clarity, we denote $I_{k}$ as the protein indicator for the *k*th read. An example is provided in the Appendix, available as supplementary data at *Bioinformatics Advances* online to further illustrate the concept of *I*. Both $I_{k}$ and $F_{k}^{o}$ are hidden variables, while $X_{k}$ is observable. The relationship between these variables is illustrated in Fig. 1.

Each probability $q_{y}$ is related to the original protein distribution $P_{Y}(y)$ as:


(3) $$q_{y}=P_{I}(y)=KE_{F^{o}}(y)P_{Y}(y)=KE_{F^{o}}(y)p_{y}$$

where $E_{F^{o}}(y)$ is the average number of observable fluorescence strings produced by protein *y*, and *K* is a normalizing factor ($K^{-1}=\sum_{y\in D_{Y}}\left[E_{F^{o}}(y)P_{Y}(y)\right]$). Equation (3) shows that knowledge of $P_{Y}$ uniquely determines $P_{I}$, and also knowing $P_{I}$ allows for the reconstruction of $P_{Y}$, and an example is provided in the Appendix, available as supplementary data at *Bioinformatics Advances* online to further clarify this expression.

Next, the distribution of experimental fluorescence strings for a given set of protein abundances can be expressed using the above introduced terms. This formulation is essential for generating the datasets used in testing:


(4) $$P_{F^{o}}(f)=\sum_{y=1}^{N_{P}}P_{F^{o}I}(f,y)=\sum_{y=1}^{N_{P}}P_{F^{o}|I}(f|y)P_{I}(y)$$

where $P_{I}(y)$ is obtained from the protein distribution with (3) and $P_{F^{o}|I}(f|y)$ is known. Finally, we define $\hat{q}={\hat{P}}_{I}(y)$ as the estimated probabilities of the distribution of protein indicators.

In summary, the protein indicator *I* is a hidden random variable with a realization for each observed read. This setup allows us to formulate an EM algorithm to maximize the likelihood of the observed data and estimate ${\hat{P}}_{I}$. After fitting, the final step is to apply the inverse transformation from (3) to obtain the estimated ${\hat{P}}_{Y}$.

### 2.4 Expectation-maximization iteration

The EM algorithm iteratively increases the data likelihood to obtain local maximum-likelihood estimates. We apply EM to update the protein distribution from ${\hat{P}}_{Y}$ to ${\hat{P}}_{Y}^{\prime }$ such that


(5) $$P_{X}(x|{\hat{P}}_{Y}^{\prime })\geq P_{X}(x|{\hat{P}}_{Y}).$$

Because $P_{Y}$ uniquely determines $P_{I}$ via (3), the EM iteration can be rewritten as:


(6) $$P_{X}(x|{\hat{q}}^{\prime })=P_{X}(x|{\hat{P}}_{I}^{\prime })\geq P_{X}(x|{\hat{P}}_{I})=P_{X}(x|\hat{q})$$

where $\hat{q}$ represents the parameters of the current estimated distribution ${\hat{P}}_{I}$ and ${\hat{q}}^{\prime }$ represents the parameters of the updated estimate ${\hat{P}}_{I}^{\prime }$. The inequality in (6) is guaranteed by our algorithm (proof in Leijon and Henter 2012). The parameter update equation (derived in the Appendix, available as supplementary data at *Bioinformatics Advances* online) is


(7) $${\hat{q}}^{\prime }{\;}_{y}=\frac{1}{N_{r}}\sum_{k=1}^{N_{r}}\gamma_{y,k}=\frac{1}{N_{r}}\sum_{k=1}^{N_{r}}\frac{P_{X_{k}|I_{k}}(x_{k}|y){\hat{q}}_{y}}{\sum_{l=1}^{N_{P}}P_{X_{k}|I_{k}}(x_{k}|l){\hat{q}}_{l}}.$$

Intuitively, $\gamma_{y,k}$ can be seen as a joint probability between the *k*th read $X_{k}$ and the relative protein abundance of a protein *y*: $I_{k}$, when assuming the abundance of proteins $\hat{q}$ in $I_{k}$. Then averaging over the reads in (7) results in new estimate of the protein indicator distribution: ${\hat{q}}^{\prime }$. Updating from $\hat{q}$ to ${\hat{q}}^{\prime }$ therefore shifts the protein proportions toward those that consistently explain the observed reads.

The likelihoods $P_{X_{k}|I_{k}}(x_{k}|y)$ can be expressed in terms of the intermediate random variable $F_{k}^{o}$ (derivation in the Appendix, available as supplementary data at *Bioinformatics Advances* online), yielding


(8) $$P_{X_{k}|I_{k}}(x_{k}|y)=\sum_{f\in D_{F}(y)}P_{X_{k}|F_{k}^{o}}(x_{k}|f)P_{F_{k}^{o}|I_{k}}(f|y)$$

### 2.5 Using the fluorescence string classification for the EM iteration

Algorithms such as Whatprot and Probeam allow us to estimate the distribution of fluorescence strings for a given read, but we use posterior likelihoods in EM. For any classifier that assumes equally distributed fluorescence strings ($P_{F_{k}^{o}}^{u}(f)$) and provides a posterior estimate ${\hat{P}}_{F_{k}^{o}|X_{k}}(f|x_{k})$, we can express


(9) $$\begin{array}{c} {\hat{P}}_{F_{k}^{o}|X_{k}}(f|x_{k})=\frac{{\hat{P}}_{X_{k}|F_{k}^{o}}(x_{k}|f)P_{F_{k}^{o}}^{u}(f)}{\sum_{g\in D_{F}}{\hat{P}}_{X_{k}|F_{k}^{o}}(x_{k}|g)P_{F_{k}^{o}}^{u}(g)}\\ =\frac{{\hat{P}}_{X_{k}|F_{k}^{o}}(x_{k}|f)}{\sum_{g\in D_{F}}{\hat{P}}_{X_{k}|F_{k}^{o}}(x_{k}|g)} \end{array}$$

where the denominator can be written as a normalizing constant $C_{k}$. Therefore ${\hat{P}}_{X_{k}|F_{k}^{o}}(x_{k}|f)=C_{k}{\hat{P}}_{F_{k}^{o}|X_{k}}(f|x_{k})$. This result is important because it allows us to use the classifier’s estimates to compute $P_{X_{k}|F_{k}^{o}}(x_{k}|f)$ for the protein inference. The multiplicative term $C_{k}$ is canceled for protein inference. Using this result and (8) we can rewrite the EM update of (7) as


(10) $$\begin{array}{c} {\hat{q}}^{\prime }{\;}_{y}=\frac{1}{N_{r}}\sum_{k=1}^{N_{r}}\frac{\eta_{y,k}{\hat{q}}_{y}}{\sum_{l=1}^{N_{P}}\eta_{l,k}{\hat{q}}_{l}}\\ \eta_{y,k}=\sum_{f\in D_{F}(y)}{\hat{P}}_{F_{k}^{o}|X_{k}}(f|x_{k})P_{F_{k}^{o}|I_{k}}(f|y) \end{array}$$

This equation shows how the updated protein distribution parameters ${\hat{q}}^{\prime }{\;}_{y}$ are obtained by summing over the reads and using the posterior probabilities from the classifier to update the EM steps.

### 2.6 Optimizing memory and processing time with sparsity on the fluorescence string classification

Parameter updates at proteome scale are costly. We exploit the sparsity of the posteriors ${\hat{P}}_{F_{k}^{o}|X_{k}}(f|x_{k})$: while there may be a large number of possible fluorescence strings (approximately $15010^{3}$ for the human proteome), only a small subset typically has non-negligible likelihood for any given read.

We model this sparsity as follows:


(11) P^Fko|Xks(f|xk)=P^Fko|Xk(f|xk)δGk(f)+rkδGk(f)={1f∈Gk0f∈Gk.

The set $G_{k}$ contains the $N_{b}$ fluorescence strings with the highest posterior probabilities for the read *k*. The indicator function therefore selects only the highest posterior probabilities for each read *k*.

The normalization constant $r_{k}$:

$r_{k}=|D_{F}{|}^{-1}\left(1-\sum_{f}{\hat{P}}_{F_{k}^{o}|X_{k}}(f|x_{k})\delta_{G_{k}}(f)\right)$ ensures that the remaining probability mass is uniformly distributed among all possible fluorescence strings. This sparsification strategy significantly reduces the computational and memory requirements for estimating $\eta_{y,k}$.

Since we only need to consider fluorescence strings that are both produced by a given protein *y* and among the top $N_{b}$ estimated posterior probabilities for a read *k*, we define the set $D_{F}(y,k)=\{f\in (D_{F}(y)\cap G_{k})\}$ as the fluorescence strings that must be considered when approximating $\eta_{y,k}$. The sparsified form of the $\eta_{y,k}$ computation becomes:


(12) $$\begin{array}{c} {\hat{q}}^{\prime }{\;}_{y}=\frac{1}{N_{r}}\sum_{k=1}^{N_{r}}\frac{\eta_{y,k}^{s}{\hat{q}}_{y}}{\sum_{l=1}^{N_{P}}\eta_{l,k}^{s}{\hat{q}}_{l}}\\ \eta_{y,k}\approx \eta_{y,k}^{s}=r_{k}+\sum_{f\in D_{F}(y,k)}{\hat{P}}_{F_{k}^{o}|X_{k}}(f|x_{k})P_{F_{k}^{o}|I_{k}}(f|y). \end{array}$$

After comparing different sparsification and normalization schemes (see Appendix, available as supplementary data at *Bioinformatics Advances* online), the variant presented above showed the best performance empirically on small protein datasets. The behavior might differ at larger scales, but it does effectively reduce computational burden.

### 2.7 Posterior estimates

This section describes the different methods to obtain estimates of the posterior probabilities ${\hat{P}}_{F_{k}^{o}|X_{k}}(f|x_{k})$

### 2.8 Oracle

For benchmarking, we use an oracle posterior with access to the true fluorescence string $f_{k}^{t}$ for the *k*th read. With error rate *e*, it assigns probability 1−e to $f_{k}^{t}$ and spreads *e* uniformly over all fluorescence strings:


(13) $${\hat{P}}_{F_{k}^{o}|X_{k}}^{o}(f|x_{k})=(1-e)\delta_{f_{k}^{t}}(f)+\frac{e}{\left|D_{F}\right|}$$

When e=0, the oracle is a perfect classifier and works as an upper bound on downstream abundance accuracy. For example, if the error-free oracle fails to yield accurate protein abundance estimates under certain experimental configurations (number of Edman degradation cycles, labeling strategies, and protease choices), then real-world posterior estimates, which inevitably introduce error, are unlikely to perform adequately under those conditions.

### 2.9 Whatprot

Whatprot (Smith *et al*. 2023) is a two-stage classifier: a k-nearest neighbors step narrows candidate fluorescence strings, followed by a Hidden Markov Model (HMM) that computes the read likelihood given the fluorescence string. While the HMM in Whatprot allows for direct computation of $P_{X_{k},F_{k}^{o}}(x_{k}|f)$, doing so for every possible fluorescence string remains computationally expensive. We used Whatprot to validate the accuracy of Probeam’s predictions and subsequently used Probeam for the protein inference tasks, as its code allowed straightforward output of full posterior distributions.

### 2.10 Probeam

Probeam (Kipen and Jaldén 2023) is a faster alternative for fluorescence string classification, using a novel state-space and beam search to estimate posteriors. It trades modest accuracy for substantial speed gains.

Unlike Whatprot, Probeam does not model the blocking effect (Smith *et al*. 2024); therefore, we excluded blocking from our simulations. We made this choice for computational efficiency and workflow compatibility: Probeam enables substantially faster peptide-level inference, and the outputs could be easily modified to run the protein inference.

We acknowledge that omitting blocking can affect classification accuracy; fully incorporating it, either by extending Probeam or adapting Whatprot’s outputs, would require significant engineering effort and is beyond the scope of this work. Initial investigations show that the accuracy of Whatprot decreases from 9.8% to 7.4% in the peptide inference when considering the blocking effect; we expect Probeam to exhibit a similar behavior. If the effect was incorporated, it would not alter significantly the conclusions of this work.

### 2.11 Error metric

A standard approach for assessing parameter improvement in an EM implementation is to track the log-likelihood $P_{X}(x|\hat{q})$ across successive iterations, but this is computationally expensive in our setting. Instead, we report a distributional distance: the MAE, chosen for its simplicity and ease of interpretation since lower values directly indicate a better fit:


(14) $$\text{MAE}({\hat{P}}_{Y})=\frac{1}{N_{P}}\sum_{y=1}^{N_{P}}\left|{\hat{P}}_{Y}(y)-P_{Y}(y)\right|$$

## 3 Results

### 3.1 Datasets

Simulated datasets were generated using the Whatprot framework (Smith *et al*. 2023) to assess our algorithm’s performance. We used simulations to verify our method since real proteome-scale experimental data is not yet available, and simulations provide the necessary ground-truth protein distributions for comparison, which would be extremely challenging to obtain under real-world conditions.

For both the five-protein and whole-proteome settings, we generated ten datasets with distinct abundance profiles by sampling $N_{P}$ independent and identically distributed exponential variables and normalizing to obtain valid distributions. Throughout this paper, “protein” refers to gene-level entries in the proteome database. In the proteome case, this produces a dynamic range in which the most abundant protein is typically $∼10^{6}$ times more abundant than the least abundant.

For each experiment we created a shared pool of reads per fluorescence string (1000 for the five-protein case; 100 for the proteome case). Each of the ten datasets was then formed by sampling from this pool according to the fluorescence-string distribution in (4). The error metric used to assess performance was Mean Absolute Error (MAE), defined in (14); plots show mean MAE across datasets as points, with error bands indicating standard deviation.

Throughout the Results section, we refer to the output of peptide-level classifiers such as Whatprot and Probeam as peptide inference. While this output is formally defined in the Methods section as fluorescence string classification, we adopt the term peptide inference here for clarity and to remain consistent with terminology commonly used in related literature. We also used an oracle-based peptide inference method to set a lower bound, and a uniform guess over the protein estimations as the upper bound.

We used Probeam to infer the posterior probabilities of peptides for each read, as described in (9). This choice was primarily motivated by the flexibility of Probeam’s in-house implementation, which allowed us to modify the code to output the top $N_{b}$ peptides rather than only the most likely one. Although prior work suggests that Probeam performs slightly worse than Whatprot for the peptide-inference task (Kipen and Jaldén 2023), we show in the Appendix, available as supplementary data at *Bioinformatics Advances* online that both classifiers yield comparable accuracy on the datasets used in this study.

Whole-proteome runs used GPU acceleration on an NVIDIA DGX with Tesla V100 GPUs (implementation details in the Appendix, available as supplementary data at *Bioinformatics Advances* online); the five-protein experiments used a simple CPU-based Python script to facilitate reproducibility.

### 3.2 Five proteins abundance estimation

While the fluorosequencing platform is ultimately intended for whole-proteome abundance estimation, it is also well-suited for early-stage development and use cases involving smaller sets of proteins. To explore this regime, we simulate a simplified scenario comprising only five proteins (PSME4, HBA2, HBB, PSME3, and PSMB10), as might arise in the case of a mixture of isolated proteins or a highly enriched, partially purified proteomics sample.

In this experiment, we use two peptide inference strategies to compute the posterior estimates needed for EM: an oracle and a modified version of Probeam, both explained in section Posterior estimates. The error rates of the simulations were parameters used in previous publications and are specified in the Appendix, available as supplementary data at *Bioinformatics Advances* online.

### 3.3 Comparison of abundance estimation

**Figure 2:**
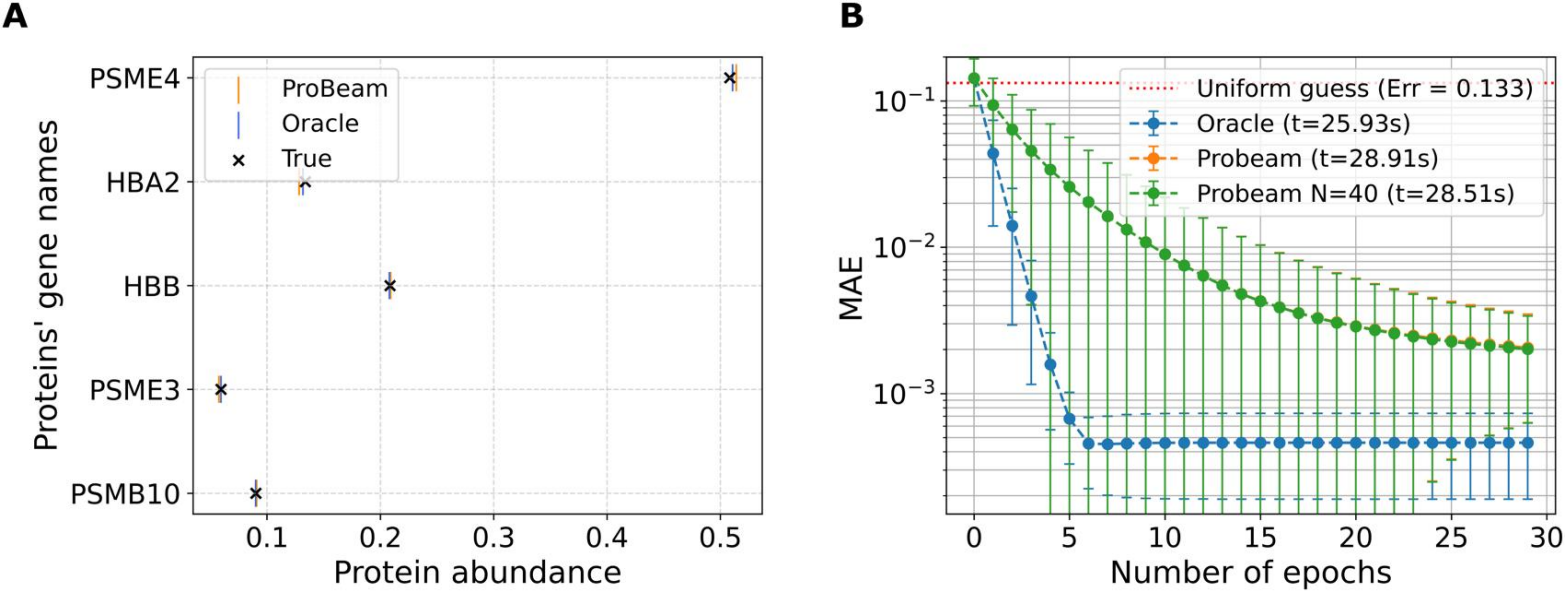
Performance comparison across different posterior estimates for a 5-protein experiment. (A) Protein abundance estimation from single peptide reads. (B) EM curves by inference method.

A direct comparison of convergence curves across all methods is presented in Fig. 2B, which includes the oracle, full Probeam, and sparse Probeam ($N_{b}=40$). The presented computing time on the legend takes into consideration the whole ten datasets. While both Probeam variants converge similarly and lag behind the oracle in terms of final accuracy, the sparse version requires less computations and memory. For five proteins the differences between the sparse and non-sparse are not significant, but the sparse method is more scalable in terms of the amount of proteins in the dataset.

Finally, Fig. 2A illustrates the ground truth versus the estimated protein abundances for each method in one of the ten datasets. All estimates closely align with the true values, confirming that even approximate posteriors derived from practical peptide inferences with currently achievable experimental errors can provide useful and reliable abundance estimates for datasets with few proteins.

In summary, this section demonstrates that the proposed EM-based inference method performs robustly in small-scale settings. For applications involving few proteins -whether for prototyping, targeted studies, or constrained biological contexts- this approach offers a practical and effective solution for protein abundance estimation.

### 3.4 Oracle performance

**Figure 3:**
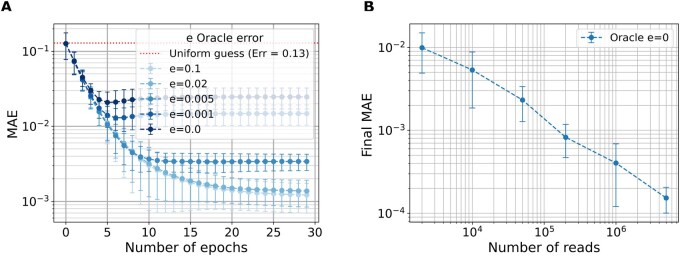
Sensitivity to oracle error rate and number of reads in a 5-protein experiment classified with an oracle. (A) Convergence curves for different oracle classification error rates *e* ($N_{r}=10^{6}$). (B) Final MAE for different $N_{r}$.

We analyze how an imperfect peptide classifier oracle with error rate *e* affects the protein inference quality. As shown in Fig. 3A, increasing the error rate leads to a marked degradation in the accuracy of the protein abundance estimates. This behavior is expected: inaccurate posterior probabilities of peptides result in larger deviations from the true protein abundance. Additionally, the convergence curves do not necessarily decrease monotonically. This is not contradictory to EM theory, which guarantees monotonically increasing data likelihood, not necessarily monotonic improvement in an external metric such as the MAE.

Next, we evaluate the impact of the number of reads on estimation accuracy, as shown in Fig. 3B. With a perfect oracle (e=0), increasing the number of reads consistently enhances the estimation performance. This behavior shows that a larger dataset corresponds to improved abundance accuracy when the peptides are perfectly distinguishable.

### 3.5 Whole proteome protein inference

This section presents an evaluation of our algorithm’s ability to estimate protein abundances at the whole-proteome scale. The dataset was constructed from the human proteome as defined in UniProt, excluding only 18 proteins that did not generate any observable tryptic peptides, resulting in *N*_p_=20 642 proteins.

### 3.6 Comparison of abundance estimation

**Figure 4:**
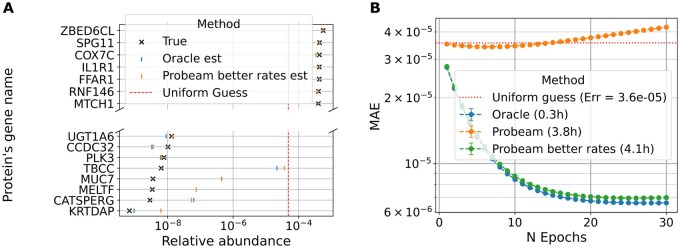
Protein inference on whole proteome ($N_{P}=20642$, $N_{r}=10^{7}$). (A) Protein abundance estimation from single peptide reads. (B) EM curves by inference method.

We now compare protein inference results obtained using the oracle and sparse Probeam ($N_{b}=1000$), under both typical and reduced experimental error conditions and $10^{6}$ reads. The sparsity value was chosen so that the computation times remained tractable, and the convergence characteristic was illustrated correspondingly. Figure 4B shows the convergence behavior of the EM algorithm for each case, with runtimes reported as the average per dataset.

Using the method described in the Appendix, available as supplementary data at *Bioinformatics Advances* online, we estimate that each of the ten datasets contains approximately $3\times 10^{5}$ protein molecules. This method also allows us to estimate the expected number of reads contributed by each protein, and we verified that, on average, 99.9% of proteins were represented by at least one read in each dataset.

The oracle achieves the lowest estimation error, establishing a best-case reference for inference performance. Under standard experimental conditions, Probeam initially performs better than random guessing; however, its estimation error increases with successive EM iterations. This degradation is consistent with the behavior observed in high-error oracle classifiers and can be attributed to the posterior estimates of Probeam not being sparse enough (see Appendix, available as supplementary data at *Bioinformatics Advances* online). To mitigate this degradation, we stop updates at the epoch that minimizes MAE, which yields small but consistent improvements over a uniform baseline. In real experimental data, this criterion is not directly available because the ground-truth abundances are unknown; thus, early stopping should be viewed as a pragmatic heuristic rather than a principled stopping rule. In practice, a reasonable stopping epoch can be calibrated from simulation studies under plausible error regimes.

When experimental error rates are artificially reduced, Probeam produces substantially more accurate estimates. In this regime, the EM algorithm converges to protein abundance distributions that more closely reflect the ground truth. The simulation parameters used to evaluate this improved performance are provided in the Appendix, available as supplementary data at *Bioinformatics Advances* online. A sensitivity analysis with respect to error rates is also presented in the Appendix, available as supplementary data at *Bioinformatics Advances* online. The oracle results can also be interpreted as the limiting case of Probeam as experimental error rates approach zero.

Finally, Fig. 4B also highlights that oracle-based inference requires significantly less computational time than Probeam. Nevertheless, our EM algorithm remains computationally efficient and can be executed at whole-proteome scale using standard GPU resources.

Figure 4A visualizes the inferred protein distribution versus the true distribution for a single dataset. In this figure, proteins are sorted by their true abundances (*y*-axis), while the *x*-axis displays the true abundance values and the corresponding EM-based estimates after thirty epochs. The results show clearly that all classifiers outperform random guessing, with the oracle providing the closest match to the ground truth. For visualization clarity, only the improved-error version of Probeam is shown. An additional visualization of this example is included in the Appendix, available as supplementary data at *Bioinformatics Advances* online.

Although relative abundance estimates are less accurate in this large-scale setting than in the five-protein case, the algorithm still provides meaningful improvement over baseline methods. Importantly, our EM framework enhances inference for any initial abundance estimate. Therefore, it can be combined with other methods to achieve a more accurate protein inference on whole-proteome scale.

### 3.7 Oracle performance

**Figure 5:**
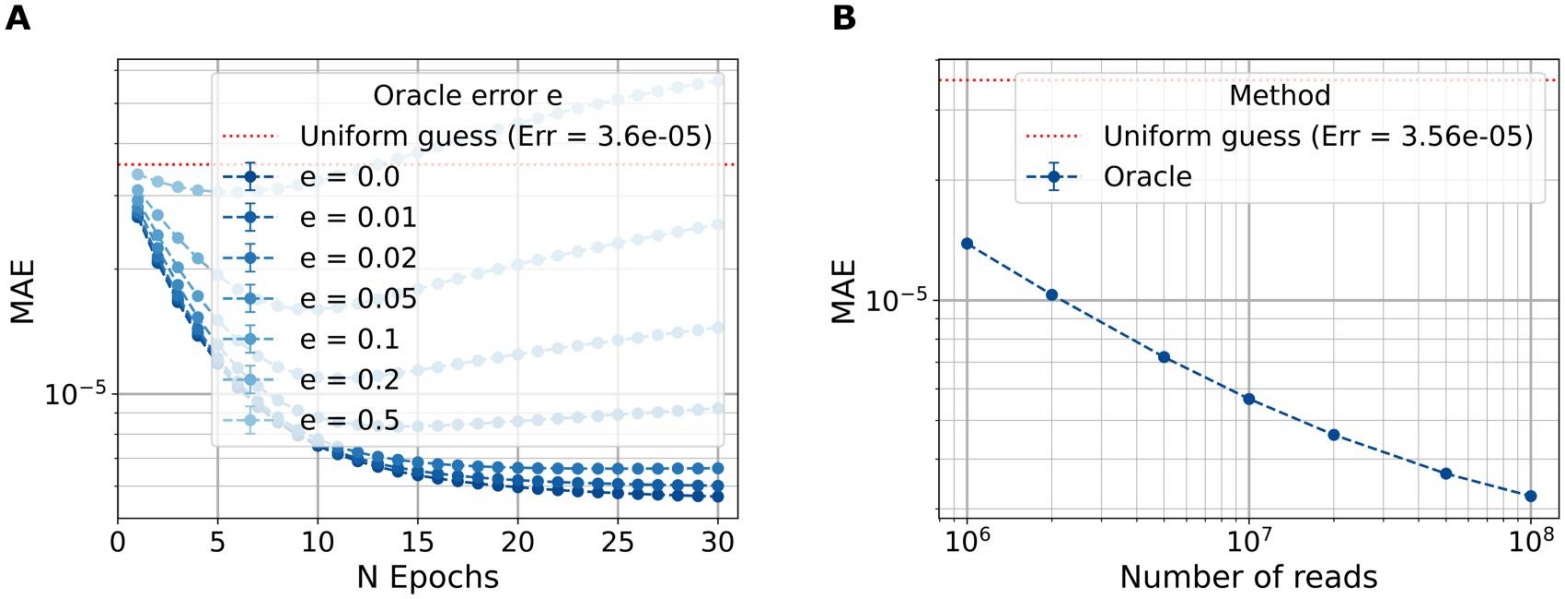
Sensitivity of the algorithm with respect to the number of reads and the classifier error rate. (A) EM for different errors on oracle ($N_{r}=10^{7}$). (B) Final MAE for different $N_{r}$.

We also analyze the performance of the oracle classifier in this large-scale setting. Figure 5B shows how the final MAE after 30 epochs varies with the number of reads, assuming a perfect peptide sequencing. Figure 5A illustrates the impact of different oracle’s classification error probabilities *e* on protein abundance estimation.

As observed in the five-protein case, increasing the number of reads improves the quality of the abundance estimates. Similarly, increasing the classification error degrades performance, with convergence curves again showing non-monotonic behavior. One key difference in this full-proteome setting is that the error bars in the plots are negligible and not visually apparent, indicating low variance across different datasets.

Overall, the perfect oracle represents an idealized case in which peptide classification is error-free. As expected, it leads to significantly more accurate abundance estimation and serves as a reference point for evaluating also protease and labeling schemes.

## 4 Discussion

We presented an EM-based protein inference framework tailored to fluorosequencing. Simulations show robust abundance estimates under realistic error rates in small-scale settings and tractable runtime at proteome scale, indicating compatibility with high throughput demands of fluorosequencing. However, this study has limitations such as its reliance on simulations in the absence of proteome scale experimental data based on a reliable ground truth, and the modest estimation improvements at proteome scale under current error rates, although it establishes a framework that can benefit directly from future improvements in chemistry and classifiers.

The scalability of our algorithm is critical for single-molecule protein sequencing, where throughput exceeds that of traditional protein sequencing methods. This approach is in principle applicable to related technologies that obtain peptide-level information, including nanopore-based methods (Brinkerhoff *et al*. 2021, Zhang *et al*. 2021). These technologies can provide peptide-level posteriors; with minor adjustments to how peptides are generated (e.g. removing the fluorosequencing-specific non-observability of dye-free fluorescence strings), our EM framework can convert their read-level outputs into protein abundance estimates.

From an accuracy standpoint, future work could explore the integration of expert prior knowledge or alternatively sourced information into the prior distributions on proteins, as is, e.g. done with TPM data in IsoBayes (Bollon *et al*. 2025). In addition, this EM-based method could serve as a refinement step for other inference pipelines by starting the EM protein inference from an initial abundance estimate rather than from a uniform protein abundance, as we did in this work. Another avenue would be to devise efficient approximations of the full data likelihood to apply a multi-start approach in the EM. Such computations would make it feasible to run EM from different initial protein distributions and, after convergence, rank the resulting solutions according to their likelihood, retaining the one that achieves the highest local maximum.

On the performance side, working on optimizing sparsification of posteriors, EM acceleration (e.g. variational or stochastic EM, quasi-Newton updates), and lightweight partial/batch updates of the protein abundances could reduce memory usage and runtime further.

Finally, the integration of deep learning into this pipeline presents a compelling opportunity. One option could be to train neural networks to predict protein abundances directly from the raw fluorescence reads, by passing some of the intermediate steps entirely. Alternatively, one could learn the η values in (10) used in the EM updates using a neural network, allowing the EM framework to function in conjunction with learned components. Both approaches could be trained with supervision, minimizing a loss with the estimation against the ground truth obtained in simulations, and potentially using architectures like Convolutional Neural Networks and attention mechanisms. Such hybrid or full data-driven approaches may not only accelerate inference but also increase robustness to modeling mismatches and experimental noise, effectively adapting the model to the data.

## Supplementary Material

vbag053_Supplementary_Data

## Data Availability

The code and data utilized to produce all the results of this paper is at https://github.com/JavierKipen/ProtInfGPU.
