# Supplementary material for: Protein abundance inference via expectation-maximization in fluorosequencing
Source: Bioinform Adv. 2026 Feb 15;6(1):vbag053. doi: 10.1093/bioadv/vbag053 (PMC12961269; doi:10.1093/bioadv/vbag053)
Supplement: vbag053_Supplementary_Data [file vbag053_supplementary_data.pdf]

Appendix

Illustrative examples of the presented random variables

This subsection presents illustrative examples to clarify the notation. Table 1 summarizes all variables and symbols.

Table 1. Variables and symbols summary

Variable or symbol	Description
$\mathcal{D}_F$	Sample space of all possible fluorescence strings.
$\mathcal{D}_Y$	Domain of proteins (gene-level entries in this work).
$\delta_S(f)$	Indicator function which returns whether the fluorescence string f is in the set S.
e	Oracle classification error rate.
$E_{\mathrm{F^o}}(y)$	Expected number of observable fluorescence strings produced by protein y.
$\eta_{y,k}$	Term used in the EM update for protein y and read k
F	Uniformly drawn fluorescence string in the experiment.
f_{null}	Null fluorescence string. F^{o} Experimentally observable fluorescence string.
I	Protein indicator (protein of origin for a read).
$\hat{P}_Y$	Estimated protein abundances.
N_{b}	Number of top posterior entries retained per read (sparsification parameter).
$N_{\mathrm{d}}(f)$	Number of ideally labeled amino acids in fluorescence string f.
N_{P}	Number of proteins in a given fluorosequencing dataset.
N_{r}	Number of reads in a given fluorosequencing dataset.
$\boldsymbol{X}$	Fluorosequencing dataset of N_r reads; $\mathbf{x}$ denotes a realization.
X_k	kth read of a fluorosequencing dataset, matrix of fluorescence intensities with rows for dye channels and columns for Edman degradation cycles.
Y	Uniformly drawn protein.

Experimental fluorophores

An example to illustrate the difference between $P_F(F = f)$ and $P_{F^{\mathrm{o}}}(F^{\mathrm{o}} = F^{\mathrm{o}})$ is the following. Suppose that for a setting of protein distribution, protein digestor and markers with fluorophore, we get $P_F(F = f)$ as:

$$P_F(F = f) = \begin{cases} f = \text{""} = f_{\mathrm{null}} & \frac{1}{3} \\ f = \text{"....1"} & \frac{1}{3} \\ f = \text{"2.0.01"} & \frac{1}{3} \end{cases} .$$

where "" symbolizes the null fluorescence string. For example, the fluorescence string "2.0.01" represents a peptide that has two dyes of color 0, and one dye each of colors 1 and 2, arranged in the order of Edman degradation cycles, with the rightmost dye being the closest to the surface of the flow cell. This notation is described in more detail in prior work Smith et al. (2023); Kipen and Jaldén (2023).

The first step to obtain $P_{F^{\mathrm{o}}}(F^{\mathrm{o}} = F^{\mathrm{o}})$ is to consider only the non-null dye sequences. We also have to consider that the dye sequences are not observable with a probability given by all the fluorophores not attaching. Since the dye miss probability was estimated in measurements to be $m = 0.25$, we obtain that the distribution is:

$$P_{F^{\mathrm{o}}}(F^{\mathrm{o}} = f) = \begin{cases} f = \text{"....1"} & 0.43 \\ f = \text{"2.0.01"} & 0.57 \end{cases} .$$

since $N_{\mathrm{d}}(\text{"....1"}) = 1$ and $N_{\mathrm{d}}(\text{"2.0.01"}) = 4$. Here we can observe that the first fluorescence string will appear less in the experiment because many times it won't have any fluorophore attached, while the second is more likely to have at least one fluorophore attached before the degradations. If they had the same amount of fluorophores, they would be equally likely.

Table 2. Protein indicator distribution example

Protein	$P_Y(Y = y)$	$E_{\mathrm{F^o}}(y)$	$P_I(I = y)$
1	0.5	7.5	0.75
2	0.5	2.5	0.25

Protein indicator distributions

Here we show in table 2 an example to illustrate the difference between P_Y and P_I:

Note that the average number of experimental fluorescence strings for a protein can be not integer. In this example, protein 1 and 2 are equally distributed, but the first one produces three times more experimental fluorescence strings than the second. This effect leads to the given distribution of the protein indicator; it is more likely that an experimentally observable fluorescence string came from the first protein than from the second.

Simulation parameters

Five proteins

The simulation parameters used for the five-protein experiments are consistent with those reported in Smith et al. (2023) and Kipen and Jaldén (2023), with one exception: the number of Edman degradation cycles was set to 11. This adjustment ensures that each fluorescence string is distinguishable by the sequencing platform.

We note that blocking probabilities (initial and cyclic) were not included in these simulations, as they are not implemented in Probeam. Nevertheless, their exclusion does not qualitatively affect the results.

Whole proteome

For the whole-proteome experiments, we simulated fluorescence reads under two distinct experimental configurations. The first reflects typical error rates observed in current fluorosequencing platforms, consistent with those used by Probeam. The second adopts significantly reduced error rates to assess inference

Table 3. Simulation parameters for the five-protein dataset.

Parameter	Value
Number of Edman cycles	11
Edman failure probability	0.06
Detach probability	0.05
Initial blocking probability	0
Cyclic blocking probability	0
Dye bleach probability (all fluorophores)	0.05
Dud probability (all fluorophores)	0.07
Mean Gaussian light intensity	10000
Standard deviation of Gaussian intensity	1600
Standard deviation of background noise	66.7

Table 4. Simulation parameters for the whole-proteome dataset under standard and improved error conditions.

Parameter	Standard Error (Probeam)	Improved Error
Number of Edman cycles	39	39
Edman failure probability	0.06	0.0006
Detach probability	0.05	0.0005
Initial blocking probability	0	0
Cyclic blocking probability	0	0
Dye bleach probability (all fluorophores)	0.05	0.0005
Dud probability (all fluorophores)	0.07	0.0007
Mean Gaussian light intensity	10000	10000
Standard deviation of Gaussian intensity	1600	160
Standard deviation of background noise	66.7	66.7

performance under near-ideal experimental conditions. All the error rates are shown in Table 4.

The number of Edman degradation cycles was set to 39 in both configurations. Shorter sequences led to substantial overlap among fluorescence strings, making many of them indistinguishable and thereby drastically reducing inference accuracy.

Sensitivity analysis

To quantify how inference accuracy depends on experimental error rates, we conducted a sensitivity analysis in which the measurement error rates were uniformly reduced by multiplicative factors of $2\times$, $5\times$, and $10\times$ relative to the standard setting. For each reduction level, we ran the EM inference for 30 epochs in the whole proteome case and report the lowest mean absolute error (MAE) attained across epochs. Figure 1 shows a monotonic improvement in accuracy as error rates decrease, with diminishing returns as performance approaches the oracle reference.

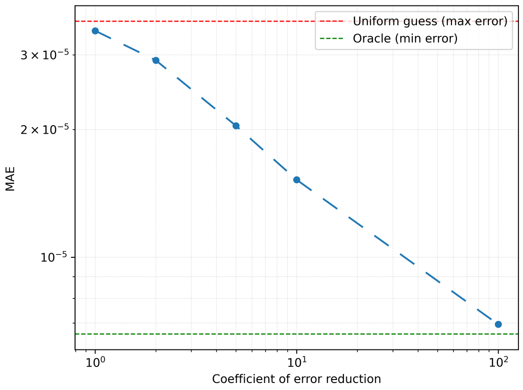

Figure 1 Sensitivity of protein inference accuracy to uniform reductions in measurement error rates.

Table 5. Comparison of Probeam and Whatprot classification accuracy across datasets and error configurations.

Number of Proteins	Error Parameters	Probeam Accuracy	Whatprot Accuracy
5	Standard	61.93%	61.94%
20,642	Standard	9.85%	9.83%
20,642	Improved	98.19%	98.15%

Probeam estimates: additional information

Comparison between Whatprot and Probeam

In this section, we compare the classification accuracy of Probeam and Whatprot to justify the use of Probeam as a surrogate for Whatprot in our experiments. Probeam was always run with 90 beams. As shown in Table 5, both methods exhibit similar accuracy across different datasets and error configurations. In some cases, Probeam even marginally outperforms Whatprot.

To observe the sparsity of the posterior estimates, we accumulate the posterior estimates ordered by their probability, and we obtain the average residue error for each N_{b} of sparsity. This is done for the three different Probeam we use and it is shown in Figures 2, 3 and 4.

To better understand the sparsity of the posterior distributions generated by Probeam, we evaluate the average residual probability as a function of the number of top N_{b} fluorescence strings retained. The x-axis represents the sparsity value, and y-axis is the average residual probability for the whole shared dataset. This analysis is presented in Figures 2, 3, and 4 for the three Probeam configurations used.

Figure 2 shows that the posterior estimates for the five-protein dataset are highly sparse, which explains why a small N_{b} still provides a good approximation. In contrast, Figure 3 reveals that even after retaining the top 1000 posterior entries, the average residue remains above 0.1. This indicates that the posterior is less concentrated, which contributes to the poorer inference performance under standard error conditions on the whole proteome. On the other hand, Figure 4 shows much lower residue values, confirming that the improved error rates result in more peaked and informative posterior distributions.

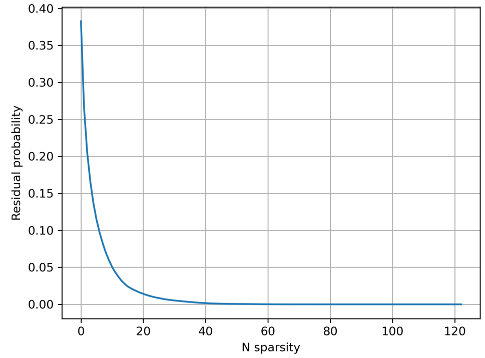

Figure 2 Average residual probability for sparse Probeam posterior estimates (standard error rates) on the five-protein dataset.

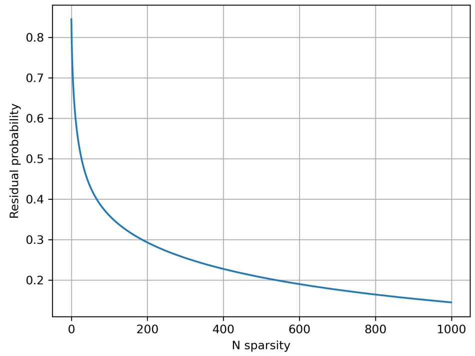

Figure 3 Average residual probability for sparse Probeam posterior estimates (standard error rates) on the whole proteome.

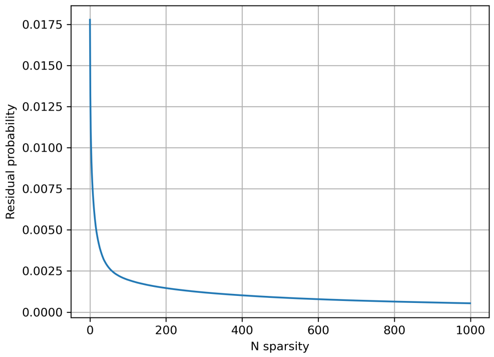

Figure 4 Average residual probability for sparse Probeam posterior estimates (improved error rates) on the whole proteome.

Additional visualization of abundance estimation

Figure 5 provides an additional diagnostic of the abundance estimator for the whole proteome. We plotted the estimated abundances using probeam with reduced rates after 30 epochs of fitting on the x-axis, against the true abundances on the y-axis. The dashed line marks $y = x$, which represents ideal estimation. Each marker compares the abundance of one single protein.

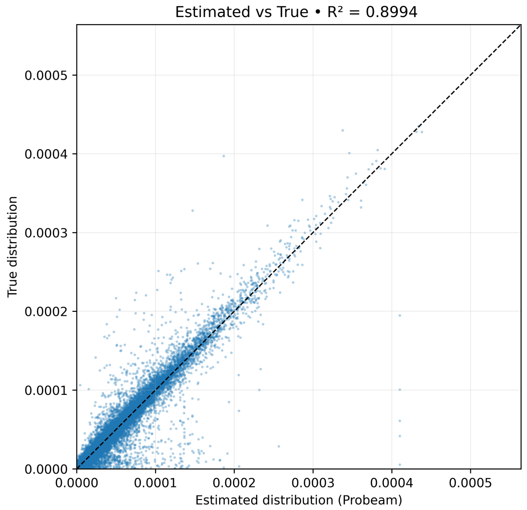

Figure 5 Scatter plot of true vs estimated protein abundances.

Table 6. Measure comparison for inference in 5-protein example

	Oracle	Probeam	Random guess
MAE	$4.62\,10^{-4}$	$2.01\,10^{-3}$	0.14
KL-Divergence	$2.17\,10^{-5}$	$1.74\,10^{-4}$	0.28

Table 7. Measure comparison for whole proteome example

	Oracle	Probeam	Random guess
MAE	$6.60\,10^{-6}$	$6.98\,10^{-6}$	$3.56\,10^{-5}$
KL-Divergence*	$3.62\,10^{-2}$	$3.95\,10^{-2}$	0.42

Overall, most points concentrate near the identity line, consistent with a high coefficient of determination R^2 and good global fit under improved error rates. A small set of visible outliers indicates remaining mismatch for certain proteins, suggesting room for further refinement.

Furthermore , we also report the Kullback-Leibler (KL) divergence alongside the MAE for the examples presented of 5 proteins and whole proteome. We use MAE as the primary metric because it is simple and, in this setting, coincides with the total-variation distance between distributions. KL divergence is a complementary measure that quantifies "distance" between distributions, and in our experiments it reflects the same qualitative trends:

*KL-divergence requires care with zeros for numerical stability; in the full-proteome setting a few probabilities reached zero, so we added a negligible constant to all likelihoods to avoid divergence. The value added to all protein likelihoods was a tenth of the lowest likelihood estimated, and then the estimation was renormalized.

Estimating protein counts

The total number of proteins in the sample, denoted K_{P}, can be estimated using the following expression:

$$K_{\mathrm{P}} \cong \frac{N_{\mathrm{r}}}{\sum_{y=1}^{N_{\mathrm{P}}} E_{\mathrm{F}^{\mathrm{o}}}(y)\, \hat{p}_y}. \tag{1}$$

where N_{r} is the total number of reads, $\hat{p}_y$ is the estimated relative abundance of protein y, and $E_{\mathrm{F}^{\mathrm{o}}}(y)$ is the expected number of observable fluorescence strings generated by protein y.

Since we do not observe which protein generated each individual read, we approximate that each protein contributes its expected number of observable reads. The protein count for a particular protein y is given by $K_{\mathrm{P}} \cdot \hat{p}_y$, and the total number of reads observed is N_{r}. By adding the expected contributions across all proteins and rearranging the terms, we arrive at Equation 1.

Applying Equation 1 to our human proteome datasets with $N_{\mathrm{r}} = 10^7$, we obtain an average estimate of $K_{\mathrm{P}} \approx 3 \times 10^5$.

Rewriting $P_{X_k|I_k}(x_k|y)$ in terms of F_k^{o}

The probabilities $P_{X_k|I_k}(x_k|y)$ can be computed using the intermediate random variable F_k^{o} as follows:

$$\begin{aligned} P_{X_k|I_k}(x_k|y) &= \sum_{f \in \mathcal{D}_{\mathrm{F}}} P_{X_k F_k^{\mathrm{o}}|I_k}(x_k, f|y) && \text{(2a)} \\ &= \sum_{f \in \mathcal{D}_{\mathrm{F}}} P_{X_k|F_k^{\mathrm{o}} I_k}(x_k|f, y)\, P_{F_k^{\mathrm{o}}|I_k}(f|y) && \text{(2b)} \\ &= \sum_{f \in \mathcal{D}_{\mathrm{F}}(y)} P_{X_k|F_k^{\mathrm{o}}}(x_k|f)\, P_{F_k^{\mathrm{o}}|I_k}(f|y) && \text{(2c)} \end{aligned}$$

In Equation 2a, the expression is equal to the joint distribution of the reads with the experimental fluorescence strings marginalized over the experimental fluorescence strings. Next, we apply the chain rule in Equation 2b. Lastly we drop the conditioning on the indicator for the first probability because of the conditional independence and we reduce the sum domain the possible fluorescence strings that can be generated by a protein y.

In the last step there are two probabilities inside the sum. First the reads likelihood is determined by the fluorescence string itself. Secondly the domain of the fluorescence strings can be reduced as the domain of fluorescence strings for a given protein y. This domain reduction significantly reduces the computational complexity, as each protein generates only a small subset of fluorescence strings.

EM iterative step proof

In this subsection, we derive the parameter update equation for the EM algorithm in fluorosequencing, which increases the full likelihood $P_{\boldsymbol{X}}(\boldsymbol{x})$, which can be rewritten to show its dependence on protein indicators:

$$\begin{aligned} P_{\boldsymbol{X}}(\boldsymbol{x}) &= \prod_{j=1}^{N_{\mathrm{r}}} P_{X_j}(x_j) \\ &= \prod_{j=1}^{N_{\mathrm{r}}} \left(\sum_{k=1}^{N_{\mathrm{P}}} P_{X_j|I_j}(x_j|I_j = k)\, P(I_j = k) \right). \end{aligned} \tag{3}$$

The function $Q(\hat{q}', \hat{q}) \triangleq E[\ln(P_{\boldsymbol{X},\boldsymbol{I}}(\boldsymbol{x}, \boldsymbol{i})|\hat{q}')|\boldsymbol{x}, \hat{q}]$ when maximized with respect to $\hat{q}'$ increases the likelihood of the reads given the new parameters $P_{\boldsymbol{X}}(\boldsymbol{x}|\hat{q}')$, as demonstrated in Leijon and Henter (2012). Here, we first rewrite the function $Q(\hat{q}', \hat{q})$, then maximize it, and finally derive the parameter update rule that results from this maximization.

To proceed, we define some useful notation. The realizations $\boldsymbol{i} = (i_1, i_2, \dots i_{N_{\mathrm{r}}})$ represents the protein indicators realizations for each read. We also introduce the vector $\boldsymbol{i}_{-k} = (i_1, i_2, \dots i_{k-1}, i_{k+1}, \dots i_{N_{\mathrm{r}}})$ which excludes the indicator for a specific read k. With this notation, we rewrite the function Q as:

$$\begin{aligned} Q(\hat{q}', \hat{q}) &= E[\ln(P_{\boldsymbol{X},\boldsymbol{I}}(\boldsymbol{x}, \boldsymbol{i})|\hat{q}')|\boldsymbol{x}, \hat{q}] && \text{(4a)} \\ &= \sum_{i_1=1}^{N_{\mathrm{P}}} \sum_{i_2=1}^{N_{\mathrm{P}}} \dots \sum_{i_{N_{\mathrm{r}}}=1}^{N_{\mathrm{P}}} P_{\boldsymbol{I}|\boldsymbol{X}}(\boldsymbol{i} = (i_1, i_2, \dots i_{N_{\mathrm{r}}})|\boldsymbol{x}, \hat{q}) \\ &\qquad \ln[P_{\boldsymbol{X},\boldsymbol{I}}(\boldsymbol{x}, \boldsymbol{i})|\hat{q}')] && \text{(4b)} \end{aligned}$$

In Equation 4, we applied the expectation definition by summing over all possible values of the hidden protein indicator vector $\boldsymbol{i}$. Next, we expand $\ln[P_{\boldsymbol{X},\boldsymbol{I}}(\boldsymbol{x}, \boldsymbol{i})|\hat{q}')]$:

$$\begin{aligned} \ln[P_{\boldsymbol{X},\boldsymbol{I}}(\boldsymbol{x}, \boldsymbol{i})|\hat{q}')] &= \ln\left[P_{\boldsymbol{X}|\boldsymbol{I}}(\boldsymbol{x}|\boldsymbol{i}, \hat{q}')\ P_{\boldsymbol{I}}(\boldsymbol{i}|\hat{q}')\right] && \text{(5a)} \\ &= \ln\left[P_{\boldsymbol{X}|\boldsymbol{I}}(\boldsymbol{x}|\boldsymbol{i})\ P_{\boldsymbol{I}}(\boldsymbol{i}|\hat{q}')\right] && \text{(5b)} \\ &= \ln\left[\prod_{k=1}^{N_{\mathrm{r}}} (P_{X|I}(x_k|i_k)\ P_I(i_k|\hat{q}'))\right] && \text{(5c)} \\ &= \sum_{k=1}^{N_{\mathrm{r}}} \ln\left[P_{X|I}(x_k|i_k)\ \hat{q}'_{i_k}\right] && \text{(5d)} \end{aligned}$$

Equation 5a follows from the chain rule and the fact that $\boldsymbol{X}$ are observations conditioned only on the hidden variables $\boldsymbol{I}$. In Equation 5b we use the independency between the different reads and that we can drop the dependance on the abundance estimations. Finally, in Equation 5c, we use the logarithm properties to make the product an external sum.

Consequently, substituting the result of Equation 5c in Equation 4b, we obtain:

$$Q(\hat{q}', \hat{q}) = \sum_{i_1=1}^{N_\mathrm{P}} \sum_{i_2=1}^{N_\mathrm{P}} \cdots \sum_{i_{N_\mathrm{r}}=1}^{N_\mathrm{P}} P_{\boldsymbol{I}|\boldsymbol{X}}(\boldsymbol{i} = (i_1, i_2, \ldots i_{N_\mathrm{r}})|\boldsymbol{x}, \hat{q}) \left(\sum_{k=1}^{N_\mathrm{r}} \ln[P_{X|I}(x_k|i_k)\, \hat{q}'_{i_k}] \right) \tag{6a}$$

$$= \sum_{k=1}^{N_\mathrm{r}} \sum_{i_1=1}^{N_\mathrm{P}} \sum_{i_2=1}^{N_\mathrm{P}} \cdots \sum_{i_{N_\mathrm{r}}=1}^{N_\mathrm{P}} P_{\boldsymbol{I}|\boldsymbol{X}}(\boldsymbol{i} = (i_1, i_2, \ldots i_{N_\mathrm{r}})|\boldsymbol{x}, \hat{q}) \ln[P_{X|I}(x_k|i_k)\, \hat{q}'_{i_k}] \tag{6b}$$

$$= \sum_{k=1}^{N_\mathrm{r}} \sum_{i_k=1}^{N_\mathrm{P}} P_{I_k|\boldsymbol{X}}(i_k|\boldsymbol{x}, \hat{q}) \ln[P_{X|I}(x_k|i_k)\, \hat{q}'_{i_k}] \tag{6c}$$

$$\sum_{i_1=1}^{N_\mathrm{P}} \cdots \sum_{i_{k-1}=1}^{N_\mathrm{P}} \sum_{i_{k+1}=1}^{N_\mathrm{P}} \cdots \sum_{i_{N_\mathrm{r}}=1}^{N_\mathrm{P}} [P_{I_{-k}|I_k,\boldsymbol{X}}(\boldsymbol{i}_{-k} = (i_1, \ldots i_{k-1}, i_{k+1}, \ldots i_{N_\mathrm{r}})|I_k = i_k, \boldsymbol{x}, \hat{q})] \tag{6d}$$

$$= \sum_{k=1}^{N_\mathrm{r}} \sum_{i_k=1}^{N_\mathrm{P}} P_{I|X}(i_k|x_k, \hat{q}) \ln[P_{X|I}(x_k|i_k)\, \hat{q}'_{i_k}] \tag{6e}$$

$$= \sum_{k=1}^{N_\mathrm{r}} \sum_{j=1}^{N_\mathrm{P}} \underbrace{P_{I|X}(j|x_k, \hat{q})}_{\gamma_{j,k}} \ln[P_{X|I}(x_k|j)\, \hat{q}'_j] \tag{6f}$$

$$= \sum_{k=1}^{N_\mathrm{r}} \sum_{j=1}^{N_\mathrm{P}} \gamma_{j,k} \left(\ln[P_{X|I}(x_k|j)] + \ln[\hat{q}'_j] \right) \tag{6g}$$

Here, in Equation 6b, we reorganize the summations. Equation 6c applies the chain rule for the indicator variable of read k (i_k), and rearranges the terms. In Equation 6e the indicator is dependent only on the kth, so the condition can be dropped from $\boldsymbol{X}$ to X_k. In Equation 6e, we exploit the fact that the indicator variable is independent of other reads, allowing us to drop the conditioning only to x_k. Finally, in Equation 6g, we arrive at the final expression for $Q(\hat{q}', \hat{q})$, which serves as the basis for deriving the EM parameter update rule.

We simplify $\gamma_{j,k}$:

$$P_{I|X}(j|x_k, \hat{q}) = \gamma_{j,k} = \frac{P_{X|I}(x_k|j, \hat{q})\, P_I(j|\hat{q})}{P_X(x_k|\hat{q})} = \frac{P_{X|I}(x_k|j)\, \hat{q}_j}{\sum_{l=1}^{N_\mathrm{P}} P_{X|I}(x_k|l)\, \hat{q}_l} \tag{7}$$

To maximize $Q(\hat{q}', \hat{q})$ over $\hat{q}'$, we need to introduce a constraint because the parameters $\hat{q}'$ have to sum up to one. Therefore, using a Lagrange multiplier λ, we define the function $L(\hat{q}', \hat{q}, \lambda)$ as:

$$L(\hat{q}', \hat{q}, \lambda) = Q(\hat{q}', \hat{q}) + \lambda \left(1 - \sum_{l=1}^{N_\mathrm{P}} \hat{q}'_l \right)$$

Taking the derivative with respect to $\hat{q}'$ and setting it to zero, we obtain:

$$0 = \frac{\partial L}{\partial \hat{q}'_j} = \frac{\partial}{\partial \hat{q}'_j} \left[\sum_{k=1}^{N_\mathrm{r}} \sum_{l=1}^{N_\mathrm{P}} \gamma_{l,k} \left(\ln[P_{X|I}(x_k|l)] + \ln[\hat{q}'_l] \right) + \lambda \left(1 - \sum_{l=1}^{N_\mathrm{P}} \hat{q}'_l \right) \right] = \sum_{k=1}^{N_\mathrm{r}} \gamma_{j,k} \frac{1}{\hat{q}'_j} - \lambda$$

Rearranging gives:

$$\hat{q}'_j = \frac{1}{\lambda} \sum_{k=1}^{N_r} \gamma_{j,k} \tag{8}$$

Since the sum of all $\hat{q}'_j$ must be 1, we solve for λ and find:

$$\lambda = \sum_{k=1}^{N_r} \sum_{j=1}^{N_p} \gamma_{j,k} = N_r$$

Equation 8 shows the final result on how to update the parameters in each EM epoch.

Sparsity sensivity for 5-protein setting

We also examine inference using posterior probabilities derived from Probeam, focusing on the role of the sparsity of peptide observations in the posterior estimates. Figure 6 shows the convergence behavior and final MAE, for different sparsity levels. In sparse approximations, low-likelihood peptides are omitted, redistributing their residual probability across all peptides. This leads to a similar degradation in accuracy as observed with oracle error, when the residual probability is considerable. Specifically, aggressive sparsification (i.e., very low N_b) results in significantly higher MAE as shown in Figure 6b, while moderate sparsity levels ($N_\mathrm{b} \geq 40$) yield estimates nearly indistinguishable from the full (non-sparse) posterior.

Sparsity analysis

We first evaluated memory usage and performance across three different sparsification strategies:

- Retaining the top N_b most likely fluorescence strings.
- Retaining the most likely fluorescence strings whose cumulative likelihood remained below a specified threshold.
- Retaining all fluorescence strings with likelihoods above a predefined threshold.

Among these approaches, the top N_b most likely fluorescence strings method achieved the best balance, significantly reducing memory usage while maintaining negligible performance loss. In contrast, the other methods required significantly more memory to achieve a similar level of performance.

For normalization, we compared the following approaches:

- No normalization.
- Scaling scores to sum to one.
- Distributing the remaining probability uniformly across all fluorescence strings.

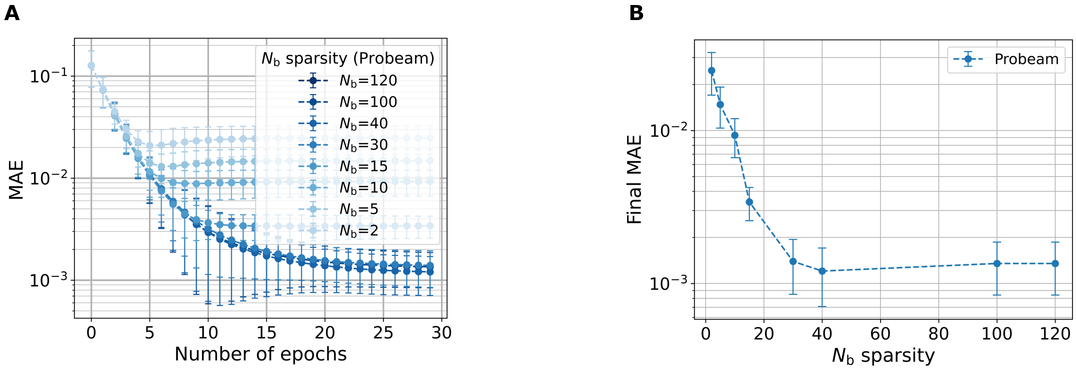

Figure 6 Effects of sparsity on convergence and final performance using Probeam for a 5-protein dataset. (A) MAE versus epochs for different peptide sparsity values. (B) Final MAE versus peptide sparsity.

- Distributing the remaining probability uniformly across all fluorescence strings not included among the top N_b sparse entries.

The best results on the five-protein datasets were obtained using the third and fourth approaches. Distributing the remaining probability uniformly across all fluorescence strings (third approach) has the additional advantage of simplifying the residual term of $\eta^{\mathrm{s}}_{y,k}$, allowing it to be factored out of the summation. The difference between the third and fourth approaches is minimal when the classifier output is already sparse and the number of possible fluorescence strings is large. For these reasons, we adopted the third approach in our experiments.

GPU implementation details

In our GPU implementation, we computed Equation $\eta^{\mathrm{s}}_{y,k}$. Since we process millions of reads simultaneously, it was infeasible to store all $\eta^{\mathrm{s}}_{y,k}$ values directly; storing this matrix would require memory equal to $N_\mathrm{r} \times N_\mathrm{P}$ floats.

To address this limitation, we allocated as much GPU memory as possible to store the largest feasible subset of $\eta^{\mathrm{s}}_{y,k}$. For the reads that fit in memory, we computed partial sums of $\eta^{\mathrm{s}}_{y,k}$ in batches, accumulating these updates across all reads. After computing the whole epoch, the complete sum is obtained and the estimated abundances are updated.

When optimizing with CuPy, we found that the sparse matrix multiplication in $\eta^{\mathrm{s}}_{y,k}$ became a major bottleneck. To overcome this, we implemented the operation directly at the CUDA level for greater efficiency. Most operations leveraged functions from the cuBLAS library; however, the sparse multiplication required a custom CUDA kernel.

After exploring different kernel designs, we settled on an approach where each block computed the sum for a subset of proteins and reads. The probabilities $P_{F^{\mathrm{o}}_k|I_k}(f|y)$ were loaded into shared memory, and each thread computed the sum for a corresponding read. We pre-sorted the indices of the sparse scores and the fluorescence strings associated with each protein. This preordering allowed faster index comparisons to identify overlapping indices between the protein and the top-scoring fluorescence strings. While the custom kernel achieved significant speed improvements, further optimization opportunities remain.

In the oracle case, where sparsity equals one, the matrix $\eta^{\mathrm{s}}_{y,k}$ is also sparse. This greatly reduced memory access requirements during writes. By optimizing this specific case, we achieved runtimes comparable to cuBLAS routines. Therefore, further optimization of this case would yield only marginal additional speed gains.

References

J. Kipen and J. Jaldén. Beam search decoder for enhancing sequence decoding speed in single-molecule peptide sequencing data. *PLOS Computational Biology*, 19(11): e1011345, 2023.

A. Leijon and G. E. Henter. *Pattern Recognition: Fundamental Theory and Exercise Problems*. School of Electrical Engineering, KTH Royal Institute of Technology, Stockholm, Sweden, 2012. 2015 ed.

M. B. Smith, Z. B. Simpson, and E. M. Marcotte. Amino acid sequence assignment from single molecule peptide sequencing data using a two-stage classifier. *PLOS Computational Biology*, 19(5):e1011157, 2023.
